# Network structure of gambling-related cognitions and risk behaviors among sports bettors in the Democratic Republic of the Congo: a cross-sectional EBICglasso network analysis (*N* = 2261)

**DOI:** 10.1016/j.abrep.2026.100746

**Published:** 2026-08-30

**Authors:** Degani Banzulu Bomba, Mechac Koffi Nkazi, Kevy Nguya Makonda, Christian Badimanya Albert, Merci Lusala Lutsasa, Jacques Benangindu Kikumpa

**Affiliations:** a Department of Psychiatry, Faculty of Medicine, University of Kinshasa, BP 127 Kinshasa XI, Democratic Republic of the Congo; b Department of Internal Medicine, Biamba Marie Mutombo Hospital, Avenue Wamba, Commune de Masina, Kinshasa, Democratic Republic of the Congo; c Department of Emergency, Intensive Care and Resuscitation, Biamba Marie Mutombo Hospital, Avenue Wamba, Commune de Masina, Kinshasa, Democratic Republic of the Congo; d Department of Emergency, Intensive Care and Resuscitation, Hôpital Général de Référence de Kinkanda, Matadi, Kongo Central, Democratic Republic of the Congo; e Department of Internal Medicine, Hôpital Général de Référence Nsona-Nkulu, Mbanza-Ngungu, Kongo Central, Democratic Republic of the Congo

**Keywords:** Gambling-related cognitions, Network analysis, Sports betting, Sub-Saharan Africa, Bridge centrality, Behavioral addiction

## Abstract

**Background and aims:** Sports betting is expanding rapidly in sub-Saharan Africa, yet the network structure of gambling-related cognitions and risk indicators remains understudied. This study analyzed these cognitions and their financial risk, psychological, and social correlates among DRC sports bettors.

**Methods:** A cross-sectional sample of 2261 sports bettors (85.9% male; mean age = 25.59 years, SD = 5.79) was recruited at Kinshasa betting venues (March–May 2026). The Gambling-Related Cognitions Scale (GRCS; α = 0.924) was administered alongside financial, psychological, and social measures. Network structure was estimated using thresholded EBICglasso (γ = 0.5). Bridge centrality and sex-based invariance were assessed via NetworkComparisonTest (100,000 permutations).

**Results:** The thresholded network retained 150 edges (density = 0.302). Guilt and G1 were the most central nodes. Bridge analysis identified guilt and social pressure as bridge nodes. Seven items showed zero bridge strength. Item-level network structure showed no global sex difference (M = 0.195, *p* = .121), whereas a 14-node sensitivity analysis detected an omnibus structural difference (M = 0.217, *p* = .022) not localized to any edge. After Bonferroni and FDR corrections, applied separately to 496 edge-level and 90 centrality-level tests, one edge (G1–G15), but no centrality index, differed by sex.

**Conclusions:** This is among the first network analyses of gambling-related cognitions in sub-Saharan Africa, with Gambling Expectancies as the most connected cognitive domain, G1 as a central hub, and guilt and social pressure as bridge nodes. These findings extend the Interaction of Person-Affect-Cognition-Execution (I-PACE) model and inform intervention research.

## Highlights

• Among the first gambling cognition network analyses in sub-Saharan Africa.
• Guilt had the highest bridge strength, linking cognitions and social pressure.
• Gambling Expectancies (GE) form the most connected cognitive domain.
• Sex-comparison findings were specification-dependent and exploratory.
• Findings extend the I-PACE (Person-Affect-Cognition-Execution) model.

## 1 Introduction

Sports betting has emerged as one of the fastest-growing forms of gambling in sub-Saharan Africa, with the Democratic Republic of the Congo (DRC) witnessing rapid proliferation of betting venues and mobile platforms, driven by smartphone penetration and youth unemployment (Bitanihirwe et al., 2022). In a six-country survey of 3879 youth aged 17–35, 54% had gambled, with estimates ranging from 42% (Ghana) to 76% (Kenya) (Ssewanyana & Bitanihirwe, 2018); lifetime sports-betting participation in East Africa has ranged from 32.3% (Kenya) to 73% (Uganda) (Bitanihirwe et al., 2022). Gambling disorder prevalence estimates vary considerably by diagnostic threshold, from approximately 0.2–0.3% for clinically significant cases under DSM-5 (American Psychiatric Association, 2013) to several percent under broader definitions. The cognitive mechanisms underlying gambling disorder in sub-Saharan Africa remain largely unexamined, and no network-based study of gambling cognitions has yet been conducted in this rapidly growing betting market. In Kinshasa, young adults often wager daily under strong peer pressure (Ssewanyana & Bitanihirwe, 2018); guilt and anxiety, commonly reported, may paradoxically reinforce betting, perhaps when losses are seen as threatening familial obligations.

The Interaction of Person-Affect-Cognition-Execution (I-PACE) model (Brand et al., 2016, 2019) posits that predisposing cognitive factors interact with affective states, executive functioning, and situational cues to maintain addictive cycles. The Gambling-Related Cognitions Scale (GRCS; Raylu & Oei, 2004) operationalizes these cognitions across five dimensions: Gambling Expectancies, Illusion of Control, Predictive Control, Inability to Stop, and Interpretive Bias. Traditional variable-centered approaches treat these dimensions as homogeneous blocks, obscuring individual items' differential roles and their connections to behavioral and emotional outcomes. Gambling-related cognitions merit attention for three reasons: they are among the most consistent correlates of gambling severity; they are modifiable, and thus the operative target of cognitive-behavioral treatment and public education; and they can be addressed through brief, low-cost, non-specialist-delivered formats realistic in settings without addiction treatment infrastructure — making a cognition-centred model the appropriate entry point here.

Network analysis offers a complementary paradigm — increasingly applied to behavioral addictions research (Fried et al., 2017; Zarate et al., 2022) — in which constructs are modeled as interacting nodes rather than reflective indicators of a latent factor (Borsboom et al., 2021; Borsboom & Cramer, 2013). Bridge nodes, at the intersection of distinct communities, serve as conduits for cross-domain activation (Jones et al., 2021). The approach offers what a latent-variable model does not: mutually interacting rather than exchangeable indicators, differential item positions, bridge analysis across a priori domains, and testable intervention priorities — though node position in a cross-sectional network remains descriptive and does not license causal inference about intervening on that node (Burger et al., 2023). The present study addresses this gap by examining the GRCS network in a context where limited regulatory frameworks, intense social pressure, and absent treatment infrastructure represent amplifiers largely absent from Western samples.

This study pursued three objectives: (1) to estimate the network structure of gambling-related cognitions, financial risk behaviors, psychological impact, and social influences using EBICglasso; (2) to identify central and bridge nodes; and (3) to test sex-based invariance. We hypothesized that (H1) Inability to Stop and Predictive Control items would show the highest centrality; (H2) nodes with high bridge centrality would connect the GRCS community to financial, psychological, and social communities; and (H3) global network structure would be invariant across sex. This study examined 2261 bettors in a non-WEIRD, sub-Saharan African setting.

## 2 Methods

### 2.1 Participants and procedure

This cross-sectional study recruited participants face-to-face at sports betting venues in Kinshasa. Trained research assistants systematically approached every third patron, explained study objectives, and obtained written informed consent. Inclusion criteria were: (a) active sports bettor status (at least one bet placed in the preceding 30 days); (b) age 18 years or older; and (c) written informed consent. Following ethical approval on March 23, 2026, data collection occurred between late March and May 2026. Games of chance and prediction contests are legally regulated in the DRC under a State licensing regime administered through the Société Nationale de Loterie (SONAL); licensed operators run street-level shops, kiosks, and mobile platforms in Kinshasa. Persons younger than 18 are prohibited from admission to gambling venues (Arrêté ministériel n° 041/MJS/CAB/2100/01/2011). The questionnaire was interviewer-administered, read aloud individually and privately rather than self-administered, to avoid literacy-related selection bias. Research assistants were physicians in postgraduate psychiatry specialty training (Diplôme d'Études Spécialisées, DES) at a university-affiliated neuropsychiatric center in Kinshasa, trained in consent procedures, neutral administration, and confidentiality. French, using the published GRCS adaptation (Grall-Bronnec et al., 2012), was the primary language; a standardized French–Lingala oral guide, reviewed by bilingual team members, was available for clarification but did not undergo independent back-translation and is not a validated Lingala version of the GRCS.

The final sample comprised 2261 participants (men: *n* = 1943, 85.9%; women: *n* = 318, 14.1%); see Table 1 and Section 3.1 for demographic and gambling-history characteristics.

**Table 1:** Descriptive Statistics for GRCS Subscales, Financial Risk Behaviors, Psychological Impact, and Social Influences (N = 2261).

**Variable**	**Total (*n*** **=** **2261)**	**Men (*n*** **=** **1943)**	**Women (*n*** **=** **318)**	**p**
Age, years	25.59 (5.79)	25.79 (5.99)	24.35 (4.15)	<0.001
Age at gambling onset, years	20.61 (5.06)	20.57 (5.22)	20.86 (3.92)	0.235

**GRCS subscales**				
Gambling Expectancies (GE)	17.36 (5.24)	17.24 (5.16)	18.05 (5.69)	0.018
Illusion of Control (IC)	12.82 (5.47)	12.74 (5.35)	13.35 (6.12)	0.096
Predictive Control (PC)	26.57 (6.31)	26.53 (6.13)	26.83 (7.32)	0.485
Inability to Stop (IS)	21.32 (7.43)	21.46 (7.26)	20.47 (8.36)	0.046
Interpretive Bias (IB)	18.43 (4.78)	18.50 (4.69)	18.01 (5.27)	0.123
GRCS Total	96.49 (24.03)	96.46 (23.41)	96.70 (27.52)	0.885

**Financial risk behaviors (% endorsing)**				
Borrowing money to gamble	55.5%	57.3%	44.0%	<0.001
Diverting funds	77.8%	80.0%	63.8%	<0.001
Lying about losses	41.8%	43.5%	31.4%	<0.001

**Psychological impact (% endorsing)**				
Anxiety	91.5%	91.9%	88.7%	0.071
Escapism	67.7%	69.3%	57.5%	<0.001
Guilt	77.7%	78.7%	71.4%	0.005

**Social influences (% endorsing)**				
Social pressure	57.7%	60.4%	41.2%	<0.001
Friends' influence				0.775
None (0)	12.8%	12.7%	13.5%	
A few (1)	60.8%	60.6%	62.3%	
Most (2)	20.7%	20.9%	19.5%	
All (3)	5.7%	5.8%	4.7%	
Online forums	78.5%	81.2%	61.9%	<0.001

**Gambling history (% endorsing / M, SD)**				
Daily gambling	68.4%	70.5%	56.0%	<0.001
Self-imposed spending limit	58.2%	59.9%	48.1%	<0.001
Betting frequency	55.8% “occasional”	57.6% “occasional”	44.3% “occasional”	<0.001
Source of gambling funds	50.7% “Other” (modal)	52.9% “others”	37.4% “others”	<0.001
Method of financing	44.1% “Income” (modal)	46.3% “income”	30.8% “income”	<0.001

*Note.* M = mean; SD = standard deviation. For binary variables, mean columns report prevalence (% endorsing). GRCS = Gambling-Related Cognitions Scale. Internal consistency (Cronbach's alpha): GE = 0.749; IC = 0.744; PC = 0.743; IS = 0.881; IB = 0.744; Total = 0.924. Sex differences were evaluated using Welch's *t*-tests for continuous variables and chi-square tests for categorical variables. Reported *p*-values are unadjusted descriptive comparisons. Daily gambling status and betting frequency were assessed as separate items with different reference framings (a direct yes/no question versus a categorical frequency scale) and are not mutually exclusive by design; the two indicators should not be read as contradictory. Percentages shown are for the most frequently reported category; full distributions are available from the corresponding author upon request. Possible ranges: Gambling Expectancies 4–28; Illusion of Control 4–28; Predictive Control 6–42; Inability to Stop 5–35; Interpretive Bias 4–28; GRCS Total 23–161.

### 2.2 Measures

#### 2.2.1 Gambling-related cognitions scale (GRCS)

The GRCS (Raylu & Oei, 2004; French version: Grall-Bronnec et al., 2012) is a 23-item measure rated on a 7-point Likert scale. It comprises five subscales: Gambling Expectancies (GE), Illusion of Control (IC), Predictive Control (PC), Inability to Stop (IS), and Interpretive Bias (IB). Higher scores indicate stronger gambling-related cognitions. Non-verbatim content descriptors for all items are provided in Supplementary Table S3. Internal consistency was excellent for the total scale (Cronbach's α = 0.924). Subscale alphas were as follows: GE = 0.749, IC = 0.744, PC = 0.743, IS = 0.881, and IB = 0.744.

#### 2.2.2 Financial risk behaviors

Three binary items (0 = absent, 1 = present) measured borrowing money to gamble, diverting funds toward gambling, and lying about gambling losses.

#### 2.2.3 Psychological impact

Three binary items assessed gambling-related anxiety, escapism, and guilt — affective variables consistent with pathways models of emotional vulnerability (Blaszczynski & Nower, 2002).

#### 2.2.4 Social Influences

Three items captured social pressure to bet (binary), friends' influence (ordinal, 0–3), and online forum participation (binary).

#### 2.2.5 Gambling History

Additional self-reported indicators captured betting frequency, daily gambling status, possession of a self-imposed spending limit, and the source and method of gambling finance.

### 2.3 Statistical analysis

**Supplementary Figure S1:**
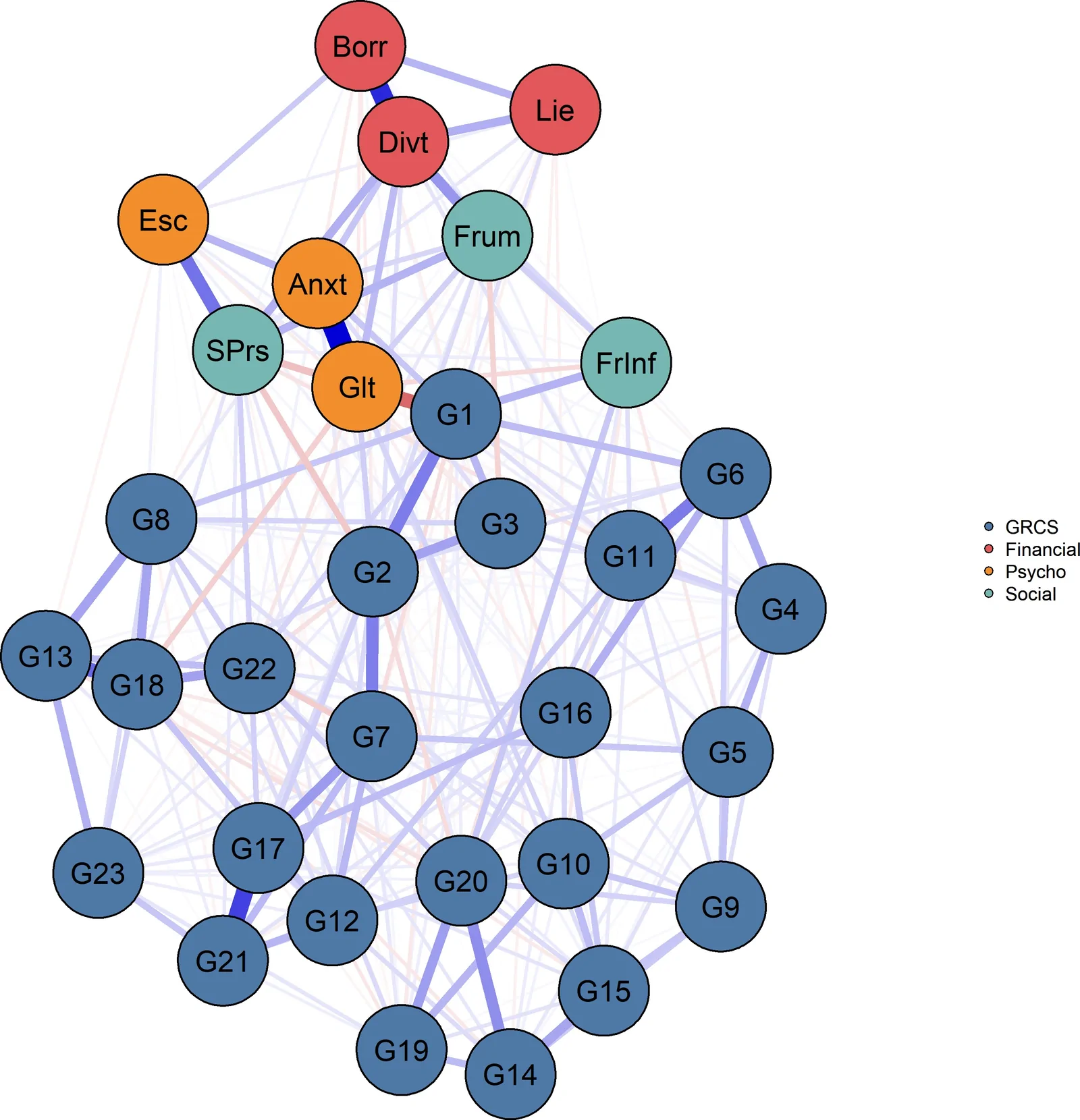
**— Unthresholded Network** Fig. S1 presents the EBICglasso network without significance threshold (289 edges, density = 0.583). Provided for transparency; the thresholded network (Fig. 1) is recommended for interpretation.

All analyses were conducted in R version 4.6.0, α = 0.05. The EBICglasso algorithm (Epskamp & Fried, 2018) was used via bootnet (v1.5), with cor_auto for mixed Likert/binary data (Epskamp et al., 2012; polychoric/tetrachoric correlations via lavaan, Rosseel, 2012) and tuning parameter γ = 0.5. A significance threshold controlled for inflated edge weights at *N* = 2261 (Isvoranu & Epskamp, 2023). Both thresholded (main) and unthresholded (Supplementary Fig. S1) networks are reported. As a sensitivity analysis, a complementary 14-node network (5 GRCS subscales +3 financial +3 psychological +3 social indicators) was estimated identically. A confirmatory factor analysis (WLSMV) of the five-factor GRCS structure was conducted using lavaan, with item allocation verified against Raylu and Oei (2004) as itemized by Kale and Dubelaar (2013).

Strength, closeness, and betweenness centrality were computed via qgraph (Epskamp et al., 2012). Stability was evaluated using case-dropping bootstrap (*n* = 1000; Epskamp et al., 2018), yielding centrality stability (CS) coefficients for each index. CS coefficients below 0.50 indicate the corresponding index should be interpreted with caution (Epskamp et al., 2018). Case-dropping bootstrap stability was also evaluated separately for men and women at both the item level and the 14-node subscale level.

Bridge strength and bridge betweenness were computed via networktools (Jones et al., 2021) across four a priori communities: GRCS items (23 nodes), financial, psychological, and social indicators (3 nodes each). Sex-based invariance was evaluated via NetworkComparisonTest (NCT; van Borkulo et al., 2023; 100,000 permutations, selected to support Bonferroni correction across the resulting tests), testing global structure (M) and strength (S) invariance. Edge-level and node-centrality *p*-values were corrected separately using Benjamini-Hochberg FDR and Bonferroni procedures.

## 3 Results

### 3.1 Descriptive statistics

The sample was predominantly male (*n* = 1943, 85.9%), with mean age 25.59 years (*SD* = 5.79; range: 18–68) and mean gambling onset 20.61 years (*SD* = 5.06). GRCS total scores ranged from 23 to 161 (Table 1). Predictive Control and Inability to Stop had the highest subscale means; Illusion of Control the lowest. Financial risk behaviors were highly prevalent: diverting funds (77.8%), borrowing money (55.5%), and lying about losses (41.8%). Psychological impact was widespread: anxiety (91.5%), guilt (77.7%), escapism (67.7%). Online forum use was reported by 78.5% and social pressure by 57.7%. Table 1 is stratified by sex. Men were slightly older (25.79 vs. 24.35 years; t(559.6) = 5.35, *p* < .001) but did not differ in gambling onset age (*p* = .235) or GRCS total (*p* = .885). Women scored higher on Gambling Expectancies (18.05 vs. 17.24, *p* = .018) and men marginally higher on Inability to Stop (21.46 vs. 20.47, *p* = .046); no other subscale differed (all *p* > .09), and none would survive multiple-testing correction. Daily gambling was more common among men (70.5% vs. 56.0%; *p* < .001), and betting frequency, spending-limit behavior, source of funds, and financing method also differed significantly by sex (all *p* < .001).

### 3.2 Network structure

#### 3.2.1 Network density and structure

**Fig. 1:**
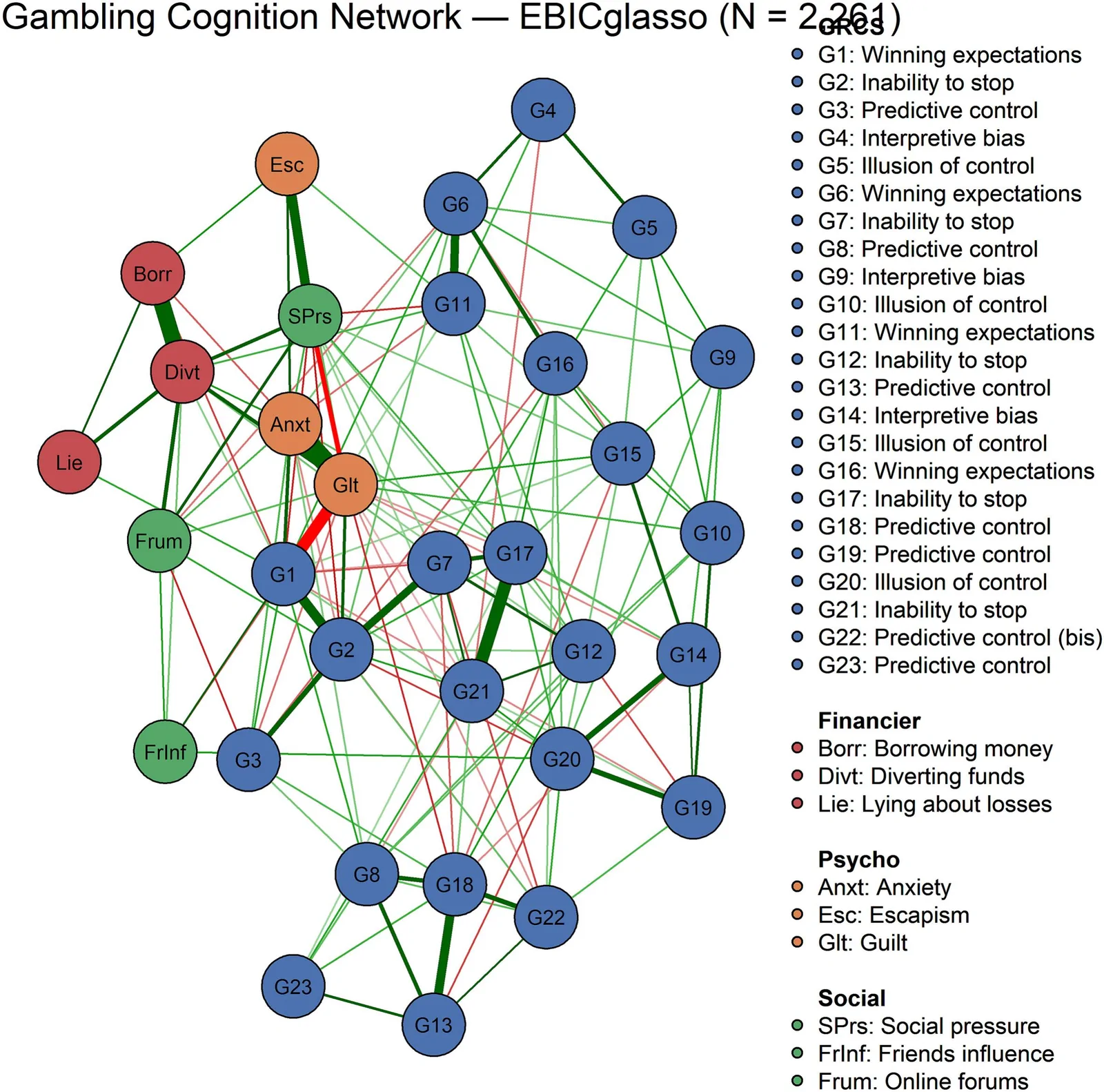
Thresholded EBICglasso network (*N* = 2261; 150 edges, density = 0.302). Node colors: blue = GRCS items (G1–G23; see Supplementary Table S3 for item content descriptors); red = financial risk behaviors (Borr = borrowing; Divt = diverting funds; Lie = lying about losses); orange = psychological impact (Anxt = anxiety; Esc = escapism; Glt = guilt); teal = social influences (SPrs = social pressure; FrInf = friends' influence; Frum = online forums). Edge thickness reflects absolute edge weight. Unthresholded network in Supplementary Fig. S1. (For interpretation of the references to colour in this figure legend, the reader is referred to the web version of this article.) Thresholded EBICglasso network (*N* = 2261; 150 edges, density = 0.302). Node colors: blue = GRCS items (G1–G23; see Supplementary Table S3 for item content descriptors); red = financial risk behaviors (Borr = borrowing; Divt = diverting funds; Lie = lying about losses); orange = psychological impact (Anxt = anxiety; Esc = escapism; Glt = guilt); teal = social influences (SPrs = social pressure; FrInf = friends' influence; Frum = online forums). Edge thickness reflects absolute edge weight. Unthresholded network in Supplementary Fig. S1. (For interpretation of the references to colour in this figure legend, the reader is referred to the web version of this article.)

The thresholded EBICglasso network (Fig. 1) contained 150 edges (density = 0.302). The unthresholded network produced 289 edges (density = 0.583; Supplementary Fig. S1). Visual inspection indicated a densely interconnected GRCS community, with guilt, social pressure, anxiety, and diverting funds emerging as cross-community connectors (see Section 3.4 for zero-bridge nodes).

#### 3.2.2 Confirmatory factor analysis of the GRCS

A confirmatory factor analysis of the canonical five-factor GRCS structure (item allocation per Raylu & Oei, 2004, as itemized by Kale & Dubelaar, 2013) showed inadequate fit in this sample: χ^2^(220) = 7976.18, χ^2^/df ≈ 36.3, CFI = 0.846, TLI = 0.823, RMSEA = 0.125 [90% CI: 0.123, 0.127], SRMR = 0.074. Given this inadequate fit, the item-level network is retained as the primary analytic unit, complemented by a 14-node subscale-level network reported as a sensitivity analysis in Section 3.5 (see Supplementary Fig. S3 for the corresponding network visualization).

### 3.3 Centrality indices

**Fig. 2:**
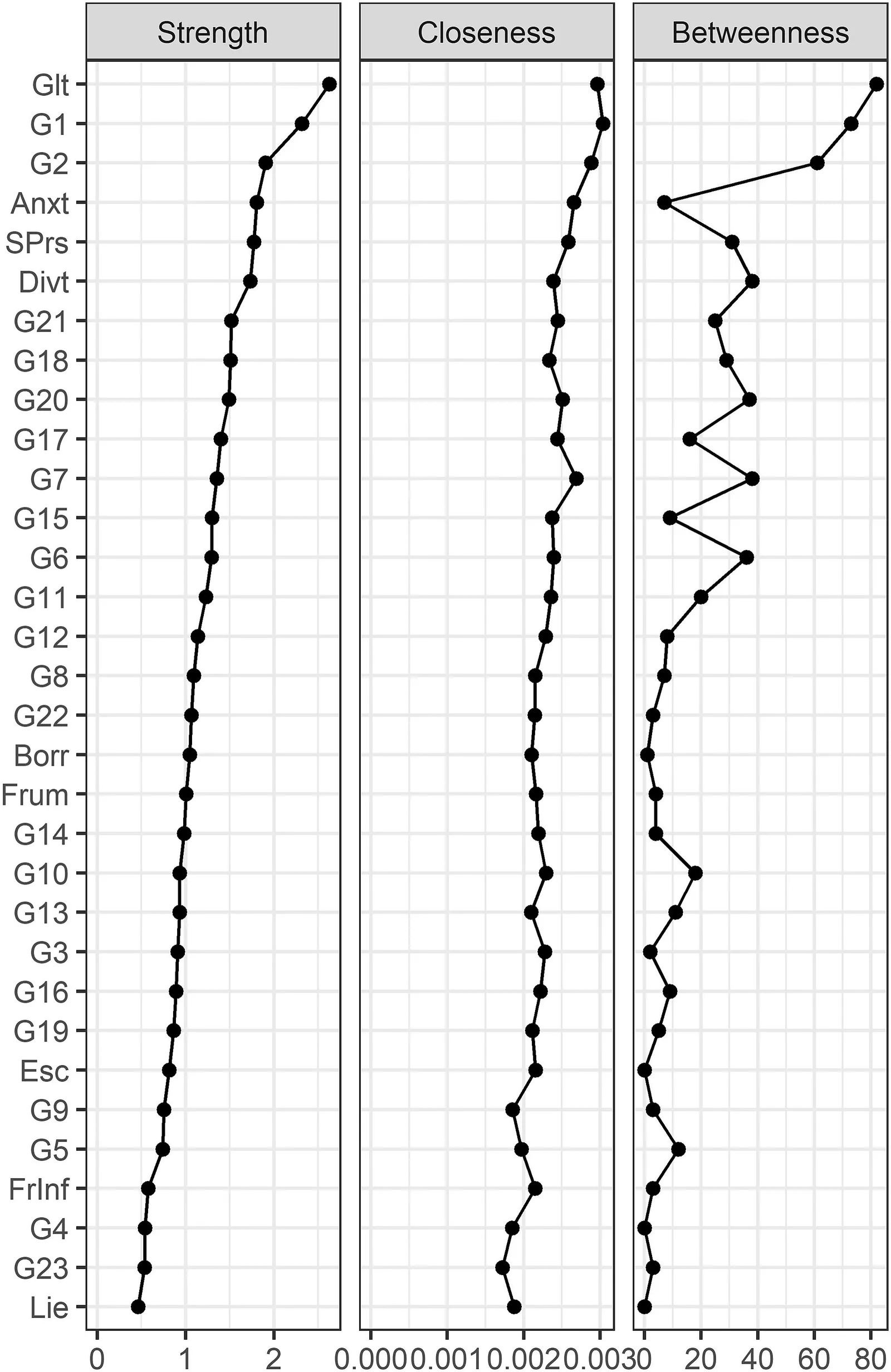
Centrality indices (strength, closeness, betweenness; standardized z-scores) for all 32 nodes ordered by strength. CS: strength = 0.672; closeness = 0.594; betweenness = 0.361.

Centrality stability analysis yielded CS coefficients of 0.672 (strength), 0.594 (closeness), and 0.361 (betweenness); given that betweenness fell below the preferred threshold, it is reported descriptively (Fig. 2, Table 2). Guilt had the highest strength (z = 2.790); G1 had the highest closeness (z = 2.332) and ranked second in strength (z = 2.180). H1 was not fully supported: several IS items (G2, G7, G17, G21) ranked among the most central nodes, whereas no PC item reached comparable centrality; guilt and anxiety also ranked highly, unpredicted by H1.

**Table 2:** Centrality Indices for All Network Nodes (Standardized z-Scores).

Node	Label	Community	Strength	Closeness	Betweenness
Guilt	Glt	*Psycho*	2.790*	2.103*	2.946*
G1	G1	*GRCS*	2.180*	2.332*	2.528*
G2	G2	*GRCS*	1.378*	1.854*	1.970*
Anxiety	Anxt	*Psycho*	1.181*	1.132*	−0.539
Social pressure	SPrs	*Social*	1.113*	0.893	0.576
Diverting funds	Divt	*Financial*	1.036	0.266	0.902*
G21	G21	*GRCS*	0.614	0.459	0.298
G18	G18	*GRCS*	0.595	0.112	0.484
G20	G20	*GRCS*	0.558	0.661	0.855
G17	G17	*GRCS*	0.378	0.436	−0.121
G7	G7	*GRCS*	0.288	1.229*	0.902*
G15	G15	*GRCS*	0.184	0.229	−0.446
G6	G6	*GRCS*	0.176	0.296	0.809
G11	G11	*GRCS*	0.049	0.179	0.065
G12	G12	*GRCS*	−0.130	−0.047	−0.492
G8	G8	*GRCS*	−0.218	−0.488	−0.539
G22	G22	*GRCS*	−0.274	−0.502	−0.725
Borrowing money	Borr	*Financial*	−0.307	−0.635	−0.818
Online forums	Frum	*Social*	−0.389	−0.456	−0.678
G14	G14	*GRCS*	−0.430	−0.347	−0.678
G10	G10	*GRCS*	−0.529	−0.030	−0.028
G13	G13	*GRCS*	−0.531	−0.654	−0.353
G3	G3	*GRCS*	−0.578	−0.077	−0.771
G16	G16	*GRCS*	−0.617	−0.265	−0.446
G19	G19	*GRCS*	−0.669	−0.598	−0.632
Escapism	Esc	*Psycho*	−0.761	−0.472	−0.864
G9	G9	*GRCS*	−0.880	−1.428	−0.725
G5	G5	*GRCS*	−0.907	−1.053	−0.306
Friends' influence	FrInf	*Social*	−1.230	−0.487	−0.725
G4	G4	*GRCS*	−1.306	−1.441	−0.864
G23	G23	*GRCS*	−1.312	−1.847	−0.725
Lying about losses	Lie	*Financial*	−1.453	−1.354	−0.864

*Note.* Values are standardized z-scores. * = top five per index. CS: Strength = 0.672, Closeness = 0.594, Betweenness = 0.361 (below preferred threshold — interpret with caution). GRCS = gambling cognition items; Financial = financial risk behaviors; Psycho = psychological impact; Social = social influences.

### 3.4 Bridge centrality

**Fig. 3:**
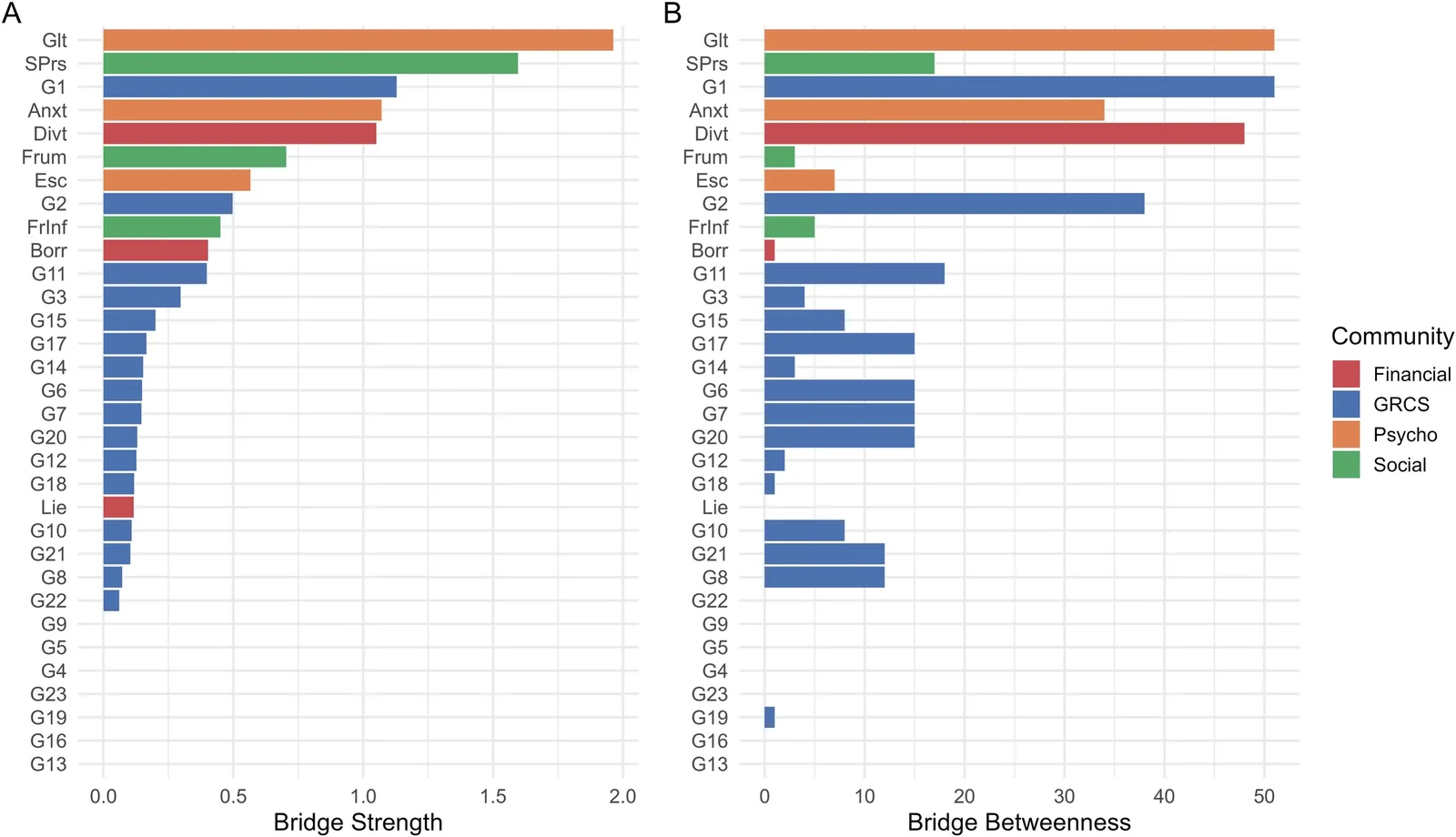
Bridge centrality indices (bridge strength and bridge betweenness) for all 32 nodes across four theoretical communities, ordered by bridge strength. Top five bridge nodes identified in Table 3.

Guilt showed the highest bridge strength (BS = 1.9624) and bridge betweenness (BB = 51), linking the psychological community to GRCS and social-influences items (Fig. 3, Table 3). Social pressure ranked second (BS = 1.5957, BB = 17). G1 showed the highest bridge betweenness (BB = 51, tied with guilt) and third-highest bridge strength (BS = 1.1285). Anxiety and diverting funds completed the top five (values in Table 3). Consistent with H2, several high-bridge-centrality nodes connected the GRCS community with financial, psychological, and social indicators. Seven GRCS items (G4, G5, G9, G13, G16, G19, G23) showed zero bridge strength (Table 3).

**Table 3:** Bridge Centrality Indices for All Network Nodes, Ordered by Bridge Strength.

Node	Label	Community	Bridge Strength	Bridge Betweenness
Guilt	Glt	*Psycho*	1.9624*	51*
Social pressure	SPrs	*Social*	1.5957*	17
G1	G1	*GRCS*	1.1285*	51*
Anxiety	Anxt	*Psycho*	1.0709*	34*
Diverting funds	Divt	*Financial*	1.0507*	48*
Online forums	Frum	*Social*	0.7038	3
Escapism	Esc	*Psycho*	0.5653	7
G2	G2	*GRCS*	0.4969	38*
Friends' influence	FrInf	*Social*	0.4501	5
Borrowing money	Borr	*Financial*	0.4024	1
G11	G11	*GRCS*	0.3975	18
G3	G3	*GRCS*	0.2970	4
G15	G15	*GRCS*	0.2003	8
G17	G17	*GRCS*	0.1654	15
G14	G14	*GRCS*	0.1525	3
G6	G6	*GRCS*	0.1489	15
G7	G7	*GRCS*	0.1459	15
G20	G20	*GRCS*	0.1298	15
G12	G12	*GRCS*	0.1264	2
G18	G18	*GRCS*	0.1179	1
Lying about losses	Lie	*Financial*	0.1163	0
G10	G10	*GRCS*	0.1079	8
G21	G21	*GRCS*	0.1037	12
G8	G8	*GRCS*	0.0720	12
G22	G22	*GRCS*	0.0605	0
G4 (zero bridge)	G4	*GRCS*	0.0000	0
G5 (zero bridge)	G5	*GRCS*	0.0000	0
G9 (zero bridge)	G9	*GRCS*	0.0000	0
G13 (zero bridge)	G13	*GRCS*	0.0000	0
G16 (zero bridge)	G16	*GRCS*	0.0000	0
G19 (zero bridge)	G19	*GRCS*	0.0000	0
G23 (zero bridge)	G23	*GRCS*	0.0000	0

*Note.* Bridge strength and bridge betweenness computed via networktools across four communities: GRCS (*n* = 23), Financial (*n* = 3), Psychological impact (*n* = 3), Social influences (*n* = 3). * = top five per index. Zero bridge strength = no cross-community edge was retained for this node under the present network specification. G1 = “Gambling makes me happier” (Gambling Expectancies item).

### 3.5 Sex-based network comparison

**Supplementary Figure S2:**
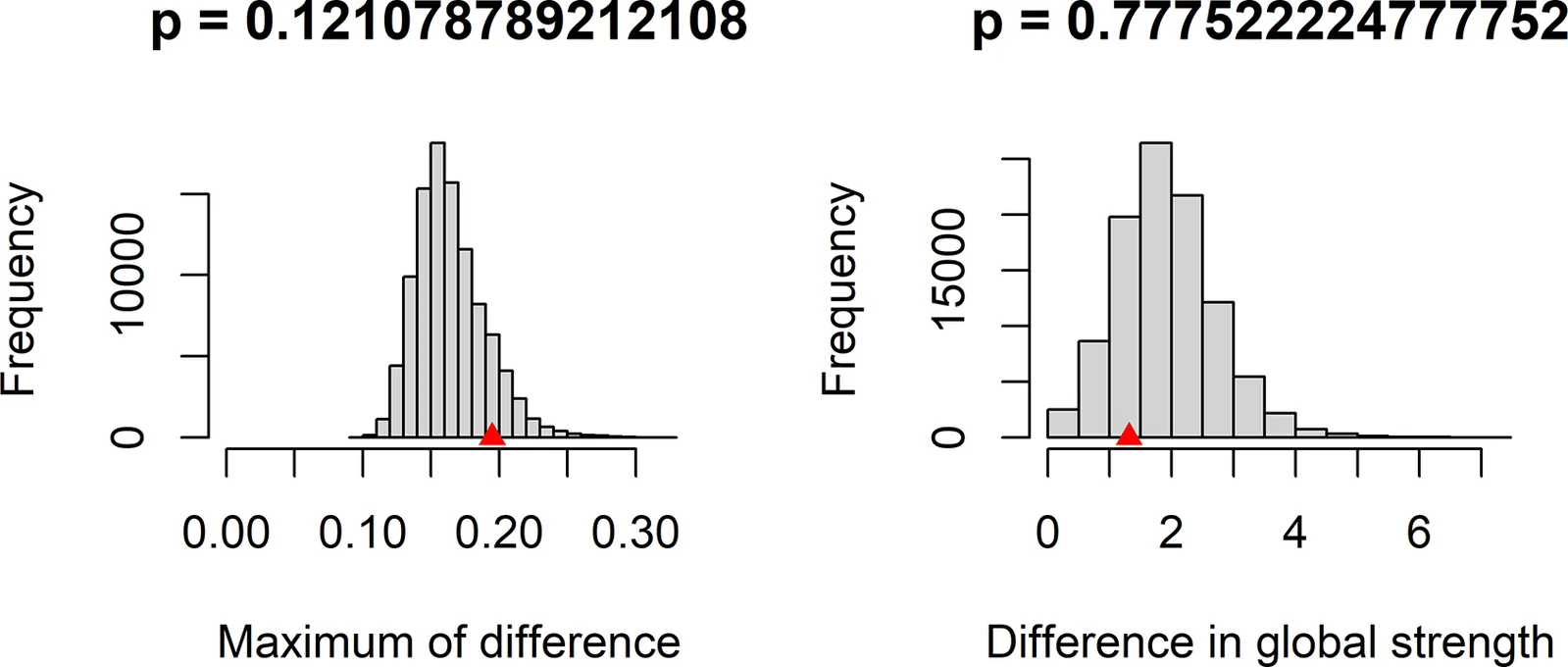
**— NCT Permutation Distributions** Fig. S2 presents permutation distributions for the two NCT omnibus tests (M and S statistics) with observed statistics as vertical lines. Men: *n* = 1943; Women: *n* = 318; permutations:100,000. Neither statistic reached significance (M: *p* = .121; S: *p* = .778).

Separate item-level networks for men (*n* = 1943; 132 edges; density = 0.266) and women (*n* = 318; 50 edges; density = 0.101) showed no significant global structural (M = 0.195, *p* = .121) or strength (S = 1.313, *p* = .778) difference. Bootstrap analysis showed the women's item-level network stability could not be estimated (non-positive-definite matrices under all configurations), precluding confident interpretation of local differences. As a sensitivity analysis, the 14-node subscale-level network was also compared by sex: structural invariance was rejected (M = 0.217, *p* = .022) while strength invariance held (S = 0.910, *p* = .449); bootstrap stability was again poor for women (CS[strength] = 0.129, below the 0.25 threshold; Epskamp et al., 2018). The NCT was re-run with 100,000 permutations to support a valid Bonferroni threshold across the 496 item-level edges. After Bonferroni and FDR correction, one item-level edge remained significant (G1–G15, linking a Gambling Expectancies item to an Interpretive Bias item; p_uncorrected <0.0001, p_Bonferroni = p_FDR = 0.005); zero edges remained significant at the subscale level (0/91). Given this combination of non-estimable or below-threshold stability with a single, narrow item-level exception and no corrected subscale-level edges, sex-based local differences are treated as exploratory rather than substantive. Full centrality and bridge centrality indices for the 14-node subscale-level network are reported in Table 4. Full details appear in Supplementary Tables S1–S2 and Fig. S2.

**Table 4:** Centrality and Bridge Centrality Indices for the 14-Node Subscale-Level Network (Sensitivity Analysis).

Node	Label	Community	Strength (z)	Bridge Strength	Bridge Betweenness
Gambling Expectancies	GE	GRCS	1.22	0.819	5
Illusion of Control	IC	GRCS	−0.40	0.393	0
Predictive Control	PC	GRCS	−0.13	0.037	2
Inability to Stop	IS	GRCS	0.10	0.445	7
Interpretive Bias	IB	GRCS	−0.10	0.259	11
Borrowing money	Borr	Financial	−0.33	0.363	0
Diverting funds	Divt	Financial	1.07	0.950	23
Lying about losses	Lie	Financial	−1.68	0.087	0
Anxiety	Anxt	Psychological	0.44	0.617	7
Escapism	Esc	Psychological	−0.92	0.519	1
Guilt	Glt	Psychological	2.02	1.393	14
Social pressure	SPrs	Social	0.46	1.156	9
Friends' influence	FrInf	Social	−1.31	0.465	0
Online forums	Frum	Social	−0.46	0.648	1

Note. Strength values are standardized z-scores; bridge strength and bridge betweenness are reported in their original metrics. Indices were calculated for the full-sample 14-node sensitivity network (*N* = 2261), comprising five GRCS subscales and nine financial, psychological, and social indicators, estimated via EBICglasso across four theoretical communities. These indices are descriptive and do not represent tests of sex differences.

## 4 Discussion

This study examined the network structure of gambling-related cognitions among 2261 sports bettors in Kinshasa, DRC — among the first such analyses in sub-Saharan Africa. Four key findings emerged: (1) a moderately sparse network (density = 0.302) with clear community organization; (2) guilt as the most central node; (3) guilt and social pressure as the leading bridge nodes; (4) no item-level difference in network structure or strength, apart from one edge (G1–G15) surviving multiple-testing correction, alongside a significant omnibus structural difference at the subscale level not localized to any individual edge.

### 4.1 Centrality: the unexpected prominence of guilt and G1

H1 was only partially supported: several IS items (G2, G7, G17, G21) ranked among the most central nodes, whereas no PC item reached comparable centrality. Guilt showed the highest strength and the second-highest closeness, while G1 (“Gambling makes me happier”; Kale & Dubelaar, 2013) — cited as a single illustrative item, consistent with the non-verbatim policy for the full scale (Section 2.2.1) — showed the highest closeness and the second-highest strength. Guilt's high endorsement (77.7%) may reflect the sample's high level of gambling involvement and prevalence of self-reported financial and psychological risk indicators. Its centrality suggests affective consequences of gambling are at least as interconnected as cognitive distortions, challenging the unidirectional cognitive-to-affective pathway of classical models (Ladouceur & Walker, 1996) and supporting a more symmetric affect-cognition role in I-PACE (Brand et al., 2019).

One possible explanation is that guilt may be especially salient where gambling losses affect both individual and family responsibilities. Network analyses of gambling cognitions in Western samples remain scarce, precluding systematic cross-cultural comparison; future studies should test whether affective nodes occupy more peripheral positions there, using continuous rather than binary guilt measures.

### 4.2 Bridge nodes: guilt and social pressure as transdiagnostic connectors

Guilt was the dominant bridge node (BS = 1.9624, BB = 51), consistent with the I-PACE feedback loop linking negative affect to compensatory cognitions in the addictive cycle (Brand et al., 2019), bridging cognitive distortions, financial behaviors, and social influences as a transdiagnostic correlate (Fried et al., 2017). Social pressure ranked second (BS = 1.5957): in the DRC, where betting venues may function as social hubs where peer norms favor risk-taking, social pressure showed cross-community associations with cognitive and behavioral indicators, highlighting the inadequacy of cognition-only interventions. G1's centrality may reflect a broadly relevant gambling expectancy, whereas the prominence of guilt and social pressure may be particularly relevant in the present sociocultural context; cross-cultural studies are needed to test this interpretation.

Seven GRCS items (G4, G5, G9, G13, G16, G19, G23) show zero bridge strength, with no retained cross-community edges under the present specification; this does not imply a distinct empirical cluster. Item codes are retained only where they add information the domain level cannot (G1; the seven zero-bridge items).

We note a methodological circularity: the item-level specification is partly motivated by the confirmatory factor model's inadequate subscale-level fit (Section 3.2.2), yet interpretations above still draw on subscale labels (GE, IC, PC, IS, IB) as organizing categories — a pragmatic but unresolved tension common in network psychometrics.

### 4.3 Sex-based network invariance

Results diverged by level of specification (Section 3.5): the item-level NCT did not detect a global difference in network structure (M = 0.195, *p* = .121) or strength (S = 1.313, *p* = .778), apart from one edge (G1–G15, linking a Gambling Expectancies item to an Interpretive Bias item) surviving correction. At the subscale level, the omnibus structure test was significant (M = 0.217, *p* = .022), although no individual edge remained significant after correction for the 91 local comparisons; given the poor stability of the women's network (CS[strength] = 0.129), this omnibus result should be considered exploratory. Taken together, these results indicate that H3 was only partially supported: sex-related differences in this network appear specification-dependent rather than demonstrating either robust invariance or a clearly localized mechanism; the sample imbalance (86% male) further constrains power for women and warrants replication in balanced samples before any clinical interpretation.

### 4.4 Theoretical and clinical contributions

We propose two candidate bridge processes that may complement existing I-PACE formulations: guilt, consistent with a possible affective-amplification role in the cognitive-behavioral feedback loop, and social pressure, consistent with a possible contextual-activation role. Mapped onto the model (Brand et al., 2016, 2019): Gambling Expectancies and Illusion of Control reflect the cognitive-bias component; guilt and anxiety, affective responses to losses; and social pressure, situational triggering cues. Financial risk behaviors represent behavioral correlates rather than a direct measure of the Execution component, which in I-PACE denotes executive functions (e.g., inhibitory control, decision-making) not directly assessed here; accordingly, this study addresses selected I-PACE processes without operationalizing the full model. Guilt's bridge position supports the updated I-PACE assumption of reciprocal affect-cognition loops. Future I-PACE formulations for sports betting in LMIC contexts should incorporate these constructs.

Clinically, three candidate priorities emerge for low-resource settings, framed as targets for future evaluation rather than demonstrated effects. First, guilt and social pressure as entry points: brief psychosocial approaches to guilt, combined with peer resistance training, may merit testing given their bridge status; both constructs may also warrant evaluation as candidate indicators for future screening or triage tools, although the present single-item measures are not validated screening instruments. Second, Gambling Expectancies, the most connected domain, as a target for public education addressing affective framing rather than probability-based messaging alone. Third, the seven zero-bridge items appear, on present evidence, lower-priority targets; whether intervening on them would alter the network cannot be determined from this cross-sectional design.

### 4.5 Limitations

Several limitations apply. First, cross-sectional design precludes causal inference. Second, convenience sampling may overrepresent frequent or highly engaged bettors, limiting generalizability to occasional, online-only, or community bettors. Third, the sex imbalance (86% male) constrains NCT power for women. Fourth, guilt and psychological variables used single binary items combined with Likert-type GRCS items; despite cor_auto's appropriate handling, item format differences may influence edge magnitudes, possibly affecting guilt's centrality. Fifth, all variables were self-reported via interviewer administration (adopted to reduce literacy-related bias), which does not permit independent verification of disclosures and may have introduced social-desirability or interviewer effects. Sixth, betweenness CS = 0.361 fell below the preferred threshold; findings are exploratory. Seventh, single-city data (Kinshasa) limit generalizability within the DRC. Eighth, the GRCS's French adaptation (Grall-Bronnec et al., 2012) has not been formally validated in a Congolese context, consistent with the inadequate CFA fit reported (Section 3.2.2); the Lingala oral guide was reviewed by bilingual staff but not independently back-translated. Ninth, the amount of money wagered or lost was not collected; financial risk is therefore captured only through the binary indicators described in Section 2.2.2, without quantifying the magnitude of exposure. Tenth, data collection spanned multiple Kinshasa betting venues (∼50 participants per venue); venue counts and refusal rates were not systematically tracked, precluding a formal response-rate estimate.

## 5 Conclusions

This study provides among the first network analyses mapping gambling-related cognitions alongside financial risk, psychological impact, and social influences in sub-Saharan Africa. Among 2261 sports bettors in Kinshasa, guilt emerged as a central bridge node, while social pressure also showed high bridge centrality, with Gambling Expectancies as the most connected cognitive domain and G1 as a central hub. The item-level omnibus test showed no global sex difference; after correction for multiple local tests, a single edge remained significant and warrants replication in balanced samples; a subscale-level sensitivity analysis detected a significant global structural difference not localized to any individual edge. These findings extend the I-PACE model by identifying guilt and social pressure as bridge mechanisms not previously modeled. Rather than a clean separation between a purely cognitive core and affective bridges, the results point to affective content — G1's expectancy of feeling happier when gambling, and guilt — occupying central positions both within and between communities, suggesting that affect and cognition are closely intertwined at the network's core. Where sports betting outpaces clinical and regulatory infrastructure, this network map provides hypotheses for the development and prospective evaluation of culturally adapted prevention and intervention strategies.

The following are the supplementary data related to this article.

**Supplementary Figure S3:**
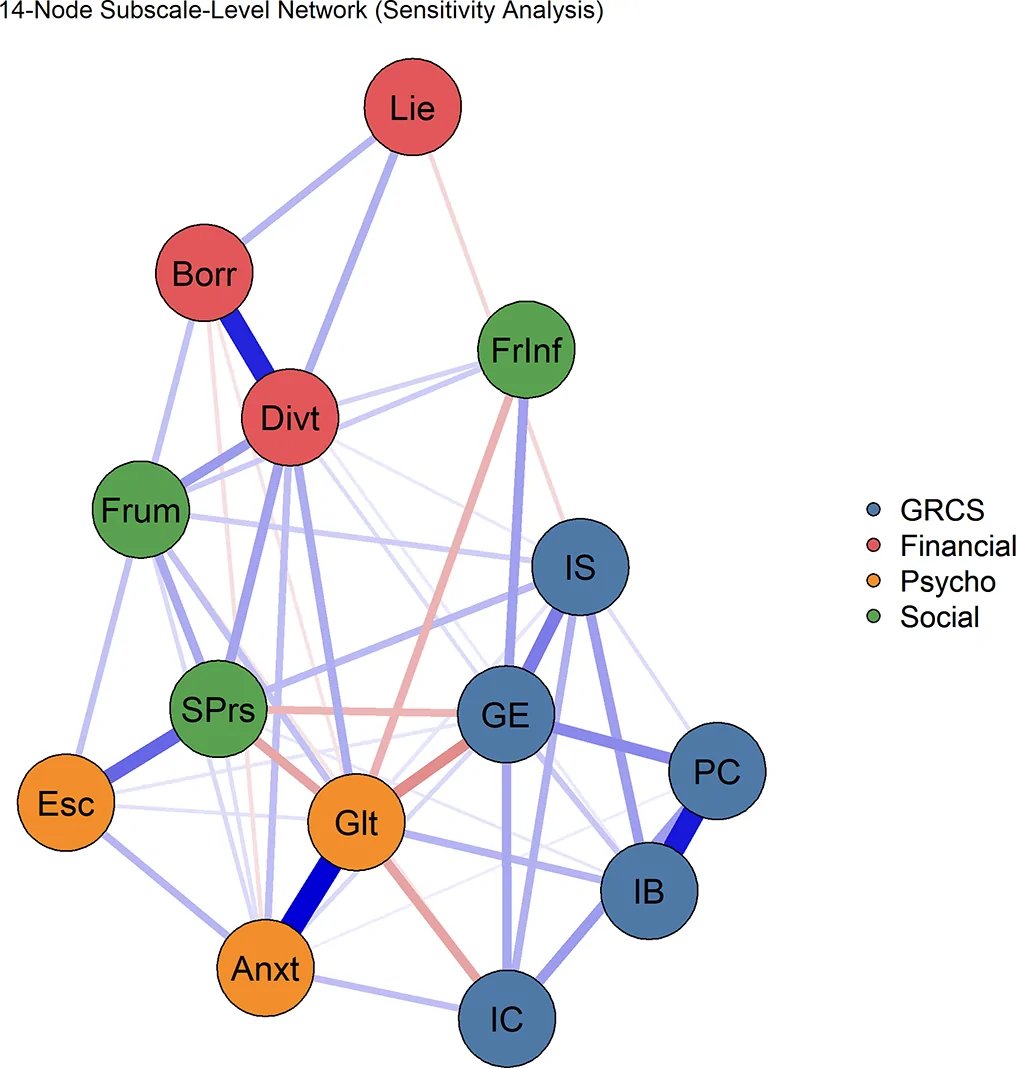
Thresholded 14-node subscale-level network (*N* = 2261; 50 edges, density = 0.549), estimated using EBICglasso with the same specification as the item-level network (Fig. 1). Node colors indicate the four theoretical communities: blue = GRCS subscales (GE, IC, PC, IS, IB); red = financial risk behaviors; orange = psychological impact; green = social influences. This sensitivity analysis complements Table 4.

Supplementary material 1 Supplementary material containing Figures S1–S3 and Tables S1–S3.

Supplementary Data 1 Complete edge-level NetworkComparisonTest results for all 496 tested item-level edges (100,000 permutations), including raw, Bonferroni-corrected, and FDR-corrected p-values. Test statistic E = absolute difference in edge weight between men (n = 1,943) and women (n = 318).

Supplementary Data 2 Complete centrality-level Network Comparison Test results for all 90 testable indices (strength, closeness, betweenness) across 32 nodes (100,000 permutations), including raw, Bonferroni-corrected, and FDR-corrected p-values. Men: n = 1,943; Women: n = 318.

## Declaration of Generative AI and AI-Assisted Technologies in the Manuscript Preparation Process

During the preparation of this work, the authors used Claude (Anthropic) in order to assist with manuscript drafting. After using this tool, the authors reviewed and edited the content as needed and take full responsibility for the content of the published article.

## CRediT authorship contribution statement

**Degani Banzulu Bomba:** Writing – review & editing, Writing – original draft, Supervision, Project administration, Methodology, Formal analysis, Conceptualization. **Mechac Koffi Nkazi:** Writing – review & editing, Investigation, Data curation. **Kevy Nguya Makonda:** Writing – review & editing, Investigation, Data curation. **Christian Badimanya Albert:** Writing – review & editing, Investigation, Data curation. **Merci Lusala Lutsasa:** Writing – review & editing, Investigation, Data curation. **Jacques Benangindu Kikumpa:** Writing – review & editing, Validation, Supervision.

## Ethics declaration

### Informed consent and patient details

Written informed consent to take part in the study and to publish the article has been obtained from all participants or their legal representatives. The privacy rights of participants have been observed.

### Studies in Human

This study was conducted in accordance with the Declaration of Helsinki. Ethical approval was granted by the Ethics Committee of the École de Santé Publique, Université de Kinshasa (reference ESP/CE/60B/2026) on March 23, 2026.

## Funding

This research did not receive any specific grant from funding agencies in the public, commercial, or not-for-profit sectors.

## Declaration of competing interest

The authors declare that they have no known competing financial interests or personal relationships that could have appeared to influence the work reported in this paper.

## Data Availability

Data are available upon reasonable request to the corresponding author.

## References

[bb0005] American Psychiatric Association (2013).

[bb0010] Bitanihirwe B.K.Y., Adebisi T., Bunn C., Ssewanyana D., Darby P., Kitchin P. (2022). Gambling in sub-Saharan Africa: Traditional forms and emerging technologies. Current Addiction Reports.

[bb0015] Blaszczynski A., Nower L. (2002). A pathways model of problem and pathological gambling. Addiction.

[bb0020] van Borkulo C.D., van Bork R., Boschloo L., Kossakowski J.J., Tio P., Schoevers R.A., Waldorp L.J. (2023). Comparing network structures on three aspects: A permutation test. Psychological Methods.

[bb0025] Borsboom D., Cramer A.O.J. (2013). Network analysis: An integrative approach to the structure of psychopathology. Annual Review of Clinical Psychology.

[bb0030] Borsboom D., Deserno M.K., Rhemtulla M., Epskamp S., Fried E.I., McNally R.J., Waldorp L.J. (2021). Network analysis of multivariate data in psychological science. Nature Reviews Methods Primers.

[bb0035] Brand M., Wegmann E., Stark R., Müller A., Wölfling K., Robbins T.W., Potenza M.N. (2019). The interaction of person-affect-cognition-execution (I-PACE) model for addictive behaviors: Update, generalization to addictive behaviors beyond internet-use disorders, and specification of the process character of addictive behaviors. Neuroscience & Biobehavioral Reviews.

[bb0040] Brand M., Young K.S., Laier C., Wölfling K., Potenza M.N. (2016). Integrating psychological and neurobiological considerations regarding the development and maintenance of specific internet-use disorders: An interaction of person-affect-cognition-execution (I-PACE) model. Neuroscience & Biobehavioral Reviews.

[bb0045] Burger J., Isvoranu A.-M., Lunansky G., Haslbeck J.M.B., Epskamp S., Hoekstra R.H.A., Blanken T.F. (2023). Reporting standards for psychological network analyses in cross-sectional data. Psychological Methods.

[bb0050] Epskamp S., Borsboom D., Fried E.I. (2018). Estimating psychological networks and their accuracy: A tutorial paper. Behavior Research Methods.

[bb0055] Epskamp S., Cramer A.O.J., Waldorp L.J., Schmittmann V.D., Borsboom D. (2012). Qgraph: Network visualizations of relationships in psychometric data. Journal of Statistical Software.

[bb0060] Epskamp S., Fried E.I. (2018). A tutorial on regularized partial correlation networks. Psychological Methods.

[bb0065] Fried E.I., van Borkulo C.D., Cramer A.O.J., Boschloo L., Schoevers R.A., Borsboom D. (2017). Mental disorders as networks of problems: A review of recent insights. Social Psychiatry and Psychiatric Epidemiology.

[bb0070] Grall-Bronnec M., Bouju G., Sébille-Rivain V., Gorwood P., Boutin C., Vénisse J.-L., Hardouin J.-B. (2012). A French adaptation of the gambling-related cognitions scale (GRCS): A useful tool for assessment of irrational thoughts among gamblers. Journal of Gambling Issues.

[bb0075] Isvoranu A.-M., Epskamp S. (2023). Which estimation method to choose in network psychometrics? Deriving guidelines for applied researchers. Psychological Methods.

[bb0080] Jones P.J., Ma R., McNally R.J. (2021). Bridge centrality: A network approach to understanding comorbidity. Multivariate Behavioral Research.

[bb0085] Kale S.H., Dubelaar C. (2013). Assessment of reliability and validity of the gambling related cognitions scale (GRCS). Gambling Research.

[bb0090] Ladouceur R., Walker M., Salkovskis P.M. (1996). Trends in cognitive and behavioural therapies.

[bb0095] Raylu N., Oei T.P.S. (2004). The gambling related cognitions scale (GRCS): Development, confirmatory factor validation and psychometric properties. Addiction.

[bb0100] Rosseel Y. (2012). Lavaan: An R package for structural equation modeling. Journal of Statistical Software.

[bb0105] Ssewanyana D., Bitanihirwe B. (2018). Problem gambling among young people in sub-Saharan Africa. *Frontiers in* Public Health.

[bb0110] Zarate D., Ball M., Montag C., Prokofieva M., Stavropoulos V. (2022). Unravelling the web of addictions: A network analysis approach. Addictive Behaviors Reports.

