# Supplementary material for: Network structure of gambling-related cognitions and risk behaviors among sports bettors in the Democratic Republic of the Congo: a cross-sectional EBICglasso network analysis (N = 2261)
Source: Addict Behav Rep. 2026 Aug 30;24:100746. doi: 10.1016/j.abrep.2026.100746 (PMC13584008; doi:10.1016/j.abrep.2026.100746)
Supplement: Supplementary file 1 [file mmc4.docx]

**Banzulu Bomba et al. — Supplementary Material**

*Network Structure of Gambling-Related Cognitions Among Sports Bettors in the DRC*

# **Supplementary Figure S1**

Figure S1. Unthresholded EBICglasso network (N = 2,261; 289 edges, density = .583). Same node colors and layout as Figure 1. Provided for transparency; the thresholded network (Figure 1, 150 edges, density = .302) is recommended for interpretation given the large sample size (Isvoranu & Epskamp, 2023).

*[Insert Figure S1 here]*

# **Supplementary Figure S2**

Figure S2 presents permutation distributions for the two NCT omnibus tests (M and S statistics) with observed statistics as vertical lines. Men: n = 1,943; Women: n = 318; permutations: 100,000. Neither statistic reached significance (M: p = .121; S: p = .778).

*[Insert Figure S2 here]*

# **Supplementary Figure S3**

Figure S3. Thresholded 14-node subscale-level network (N = 2,261; 50 edges, density = .549), estimated using EBICglasso with the same specification as the item-level network (Figure 1). Node colors indicate the four theoretical communities: blue = GRCS subscales (GE, IC, PC, IS, IB); red = financial risk behaviors; orange = psychological impact; green = social influences. This sensitivity analysis complements Table 4.

*[Insert Figure S3 here]*

# **Supplementary Table S1 — Edge-Level NCT Results (All 496 Tested Edges)**

Table S1 reports edge-level NCT results for all 496 tested item-level edges, ordered by ascending *p*-value, with uncorrected, Bonferroni-corrected, and FDR-corrected *p*-values (100,000 permutations). Forty-eight of 496 edges reached uncorrected significance (*p* < .05); only one edge (G1–G15, linking the Gambling Expectancies item “Gambling makes me happier” to an Interpretive Bias item) survived Bonferroni and FDR correction (*p*_uncorrected < .0001; *p*_Bonferroni = *p*_FDR = .005). Test statistic E = absolute difference in edge weight between men (*n* = 1,943) and women (*n* = 318). NCT = NetworkComparisonTest (van Borkulo et al., 2023).

Full results for all 496 tested edges, including raw, Bonferroni-corrected, and FDR-corrected *p*-values, are provided in **Supplementary Data 1** (R4_NCT_edges_100000perm.csv).

## **Illustrative excerpt — 10 smallest uncorrected p-values**

| **Node 1** | **Node 2** | **E** | **p (raw)** | **p (Bonferroni)** | **p (FDR)** |
| --- | --- | --- | --- | --- | --- |
| G1 | G15 | 0.0878 | <.0001 | 0.005 | 0.005 |
| G1 | G11 | 0.1568 | 0.0006 | 0.298 | 0.030 |
| G4 | G6 | 0.1907 | 0.0051 | 1.000 | 0.126 |
| G1 | G2 | 0.1949 | 0.0061 | 1.000 | 0.126 |
| G6 | Glt | 0.0430 | 0.0080 | 1.000 | 0.132 |
| G22 | Borr | 0.0204 | 0.0090 | 1.000 | 0.132 |
| G9 | G15 | 0.1756 | 0.0090 | 1.000 | 0.132 |
| Esc | Frum | 0.1229 | 0.0100 | 1.000 | 0.132 |
| G15 | FrInf | 0.0578 | 0.0100 | 1.000 | 0.132 |
| G1 | Glt | 0.1834 | 0.0130 | 1.000 | 0.153 |

*Note. Illustrative subset only, for readability; complete results for all 496 edges are in Supplementary Data 1.*

# **Supplementary Table S2 — Centrality-Level NCT Results**

Table S2 reports raw, FDR-corrected, and Bonferroni-corrected *p*-values for node-level centrality differences (strength, closeness, betweenness) across all 32 nodes (90 testable indices out of 96; closeness was non-estimable for 6 nodes — *lying about losses, anxiety, escapism, guilt, friends' influence, and online forums* — due to partial disconnection of the smaller women's network, n = 318, into three components). Men: *n* = 1,943; Women: *n* = 318. Six indices reached uncorrected significance (*p* < .05); none survived Bonferroni or FDR correction (lowest *p*_FDR = .321).

Full results for all 90 testable centrality indices are provided in **Supplementary Data 2** (R4_NCT_centrality_100000perm.csv).

# **Supplementary Table S3 — GRCS Item Content Glossary**

Table S3 lists all 23 GRCS items by code (G1–G23), subscale membership (per Raylu & Oei, 2004, as itemized by Kale & Dubelaar, 2013), and a brief, non-verbatim content descriptor for each item.

*Note. This table has been reconstructed relative to the version circulated at the previous revision round. In accordance with copyright restrictions on the published GRCS instrument and its French adaptation (Grall-Bronnec et al., 2012), the exact administered item wording is not reproduced; the descriptors below summarize item content for interpretive reference only. Researchers wishing to consult the exact item wording should refer to the original published scales.*

| **Code** | **Subscale** | **Content descriptor (non-verbatim; not the administered item wording)** |
| --- | --- | --- |
| G1 | GE | Belief that gambling improves one's mood or sense of happiness |
| G2 | IS | Perceived inability to function without gambling |
| G3 | IC | Belief that prayer influences gambling outcomes |
| G4 | PC | Expectation that a losing streak will be followed by a run of wins |
| G5 | IB | Attributing wins to one's own skill or ability |
| G6 | GE | Belief that gambling makes things seem better |
| G7 | IS | Difficulty stopping due to a perceived loss of control |
| G8 | IC | Belief that specific numbers or colors increase the odds of winning |
| G9 | PC | Belief that a losing streak provides useful lessons for future wins |
| G10 | IB | Attributing losses to bad luck or unfavorable circumstances |
| G11 | GE | Belief that gambling makes the future look more promising |
| G12 | IS | Sense that the urge to gamble is stronger than one's self-control |
| G13 | IC | Collecting objects believed to increase the odds of winning |
| G14 | PC | Belief that a win predicts further wins |
| G15 | IB | Attributing losses to probability-based reasoning that sustains continued play |
| G16 | GE | Belief that gambling reduces tension or stress |
| G17 | IS | Perceived lack of willpower needed to stop gambling |
| G18 | IC | Personal rituals or behaviors believed to increase the odds of winning |
| G19 | PC | Belief in personal “lucky” periods that determine when to gamble |
| G20 | IB | Recalling a past win as a reason to keep gambling |
| G21 | IS | Belief that one will never be able to stop gambling |
| G22 | PC | Perceived personal ability to predict gambling outcomes |
| G23 | PC | Belief that keeping the same numbers improves the odds relative to changing them |
